# Anti-Inflammatory Mechanisms of *Pleurotus citrinopileatus*: Inhibition of MAPK and NF-κB Signaling Pathways, and Activation of ROS/PI3K/Nrf2/HO-1 Signaling Pathway in LPS-Stimulated RAW264.7 Cells

**DOI:** 10.4014/jmb.2501.01012

**Published:** 2025-05-15

**Authors:** Hyeok Jin Choi, Jeong Won Choi, So Jung Park, Sang Hun Lee, Jin Hyuk Hwang, Youngki Park, Kyoung Tae Lee, Jin Boo Jeong

**Affiliations:** 1 Department of Forest Science, Gyeongkuk National University, Andong 36729, Republic of Korea; 2 Department of Forest Bioresources, Division of Forest Microbiology, National Institute of Forest Science, Suwon 16631, Republic of Korea

**Keywords:** Anti-inflammation, macrophages, mushroom, *Pleurotus citrinopileatus*

## Abstract

This study explores the anti-inflammatory potential of *Pleurotus citrinopileatus* by examining its impact on inflammation-related signaling pathways in lipopolysaccharide (LPS)-stimulated RAW264.7 macrophages. Fractions obtained using hexane (HE), dichloromethane (DCM), and ethyl acetate (EA) were found to suppress LPS-induced nitric oxide (NO) production and the expression of inducible nitric oxide synthase (iNOS). Additionally, these fractions inhibited the phosphorylation of key mitogen-activated protein kinases (MAPKs), including extracellular signal-regulated kinases 1/2 (ERK1/2), p38, and c-Jun N-terminal kinase (JNK), as well as the nuclear factor kappa B (NF-κB) subunit p65. The HE, DCM, and EA fractions also promoted the nuclear accumulation of nuclear factor erythroid 2-related factor 2 (Nrf2) and increased the expression of heme oxygenase-1 (HO-1). Notably, the suppression of HO-1 activity using zinc (II) protoporphyrin IX (ZnPP) reversed the NO-inhibitory effects of these fractions. Furthermore, treatment with the HE, DCM, and EA fractions enhanced phosphoinositide 3-kinase (PI3K) activation, whereas PI3K inhibition by LY294002 attenuated HO-1 expression and nuclear Nrf2 translocation. Reactive oxygen species (ROS) scavenging by N-acetyl-L-cysteine (NAC) similarly reduced PI3K activation and the upregulation of HO-1 and nuclear Nrf2. Collectively, these findings indicate that the HE, DCM, and EA fractions mitigate NO production by downregulating iNOS expression through the suppression of MAPK and NF-κB signaling, while also engaging the ROS/PI3K/Nrf2/HO-1 pathway to exert anti-inflammatory effects.

## Introduction

Inflammation is regarded in the human body as a complex biological response to protect against noxious stimuli, such as invading pathogens and damaged cells [1]. Therefore, inflammation serves as an essential defense mechanism critical for maintaining health [2]. However, dysregulated inflammatory responses are known to lead to chronic inflammation, contributing to the development of a wide range of chronic conditions [3].

Currently, nonsteroidal anti-inflammatory drugs (NSAIDs) represent one of the most frequently prescribed classes of drugs for managing inflammation. However, prolonged use of NSAIDs is known to pose significant gastrointestinal side effects, including mucosal lesions, bleeding, and peptic ulcers [4, 5]. Other studies have reported that NSAIDs may also induce severe complications, including acute renal failure, hypertension, and cardiovascular toxicity [6-8]. Consequently, considerable efforts have been directed toward developing plant-based anti-inflammatory compounds as potential natural therapeutics to replace NSAIDs associated with various adverse effects during long-term use [1].

Mushrooms are considered to be of significant value as a nutritional resource due to their richness in carbohydrates, proteins, free amino acids, vitamins, and a variety of essential minerals and trace elements [9, 10]. Moreover, mushrooms are reported to be abundant in bioactive metabolites such as polysaccharides, polyphenols, ergosterol, and volatile organic compounds, which contribute to their medicinal properties [11-13]. These metabolites originating from mushrooms have been identified as potential anti-inflammatory agents [1]. *Pleurotus citrinopileatu*, a member of the subphylum Basidiomycota, is recognized as a valuable economic mushroom with both edible and medicinal properties [14]. To date, numerous studies have revealed that *P. citrinopileatus* possesses a broad spectrum of bioactivities such as HIV inhibition, immune modulation, antioxidation, and prevention of metabolic syndrome [15-19]. Recently, *P. citrinopileatus* has been reported to attenuate colon damage induced by inflammatory stress, providing evidence of its anti-inflammatory activity [20]. However, studies elucidating the specific biological processes involved in mediating the anti-inflammatory response of *P. citrinopileatus* remain lacking. In this study, to elucidate the potential mechanisms underlying the anti-inflammatory activity of *P. citrinopileatus*, we further evaluated its anti-inflammatory effects and investigated its modes of action in LPS-stimulated RAW264.7 cells.

## Materials and Methods

### Chemical Reagents and Antibodies

MTT ((3-(4,5-Dimethylthiazol-2-yl)-2,5-Diphenyltetrazolium Bromide)), LY294002, Griess reagent, N-acetyl-L-cysteine (NAC), and lipopolysaccharide (LPS) were obtained from Sigma-Aldrich. Zinc (II) Protoporphyrin IX (ZnPP) was sourced from Enzo Life Sciences. Primary antibodies were purchased from Cell Signaling Technology, including those targeting extracellular signal-regulated kinases 1/2 (ERK1/2, cat. no: 9102), phosphorylated ERK1/2 (p-ERK1/2, cat. no: 4377), p38 MAPK (cat. no: 9212), phosphorylated p38 (p-p38, cat. no: 4511), c-Jun N-terminal kinase (JNK, cat. no: 9258), phosphorylated JNK (p-JNK, cat. no: 9251), NF-κB subunit p65 (cat. no: 8242), phosphorylated p65 (p-p65, cat. no: 3033), phosphoinositide 3-kinase (PI3K, cat. no: 4257), phosphorylated PI3K (p-PI3K, cat. no: 4228), heme oxygenase-1 (HO-1, cat. no: 70081), nuclear factor erythroid 2-related factor 2 (Nrf2, cat. no: 12721), and β-actin (cat. no: 5125). In addition, the secondary antibody, anti-rabbit IgG HRP-conjugated antibody (cat. no: 7074), was obtained from Cell Signaling Technology.

### Sample Preparation

The 70% methanol crude extract, hexane (HE) fraction, dichloromethane (DCM) fraction, ethyl acetate (EA) fraction, butanol (BU) fraction, and water fraction of *P. citrinopileatus* were provided by the National Institute of Forest Science, Korea. Briefly, dried *P. citrinopileatus* (50 g) was extracted at room temperature with 70% methanol at a 1:20 ratio (w/v) for 24 h. To obtain the 70% methanol extract, the extract was concentrated to 300 ml by removing the solvent under reduced pressure at a temperature below 45°C. Subsequently, sequential fractionation was performed using hexane, dichloromethane, ethyl acetate, and butanol in a 1:1 ratio (v/v). Each fraction was obtained by completely removing the solvents under reduced pressure at temperatures below 45°C. Prior to further analysis, the crude extract and its fractions were maintained at -80°C and protected from light exposure.

### Culture of RAW264.7 Cells

To evaluate the anti-inflammatory activity of *P. citrinopileatus* fractions, RAW 264.7 macrophages sourced from the American Type Culture Collection (ATCC) were employed. The cells were maintained in Dulbecco’s Modified Eagle Medium/Nutrient Mixture F-12 (DMEM/F-12, Hyclone, Cytiva) supplemented with 10% fetal bovine serum (Gibco, Thermo Fisher Scientific, Inc.), along with 100 U/ml penicillin and 100 μg/ml streptomycin. Cultures were incubated at 37°C in a humidified environment containing 5% CO_2_ and were subcultured every 2-3 days to ensure optimal growth conditions.

### Griess Assay

Nitrite (NO_2_^-^) concentration in the culture supernatant was measured to determine the inhibitory potential of *P. citrinopileatus* fractions against LPS-induced NO production in RAW 264.7 cells. RAW 264.7 cells (7.0 × 10^4^ cells/well) were plated in 24-well plates and cultured under standard culture conditions (37°C, 5% CO_2_, humidified) for 24 h. Following pre-incubation, the cells were exposed to *P. citrinopileatus* fractions along with LPS (1 μg/ml) for a further 24 h. After treatment, a 100 μl sample of the supernatant from each well was added to a 96-well plate and combined with an equal volume of Griess reagent (Sigma-Aldrich). Following incubation in the dark for 15 min, the absorbance of the reaction mixture was determined with a UV/Vis spectrophotometer (SpectraMax M2, Molecular Devices).

### MTT Assay

The MTT assay was performed to investigate potential cytotoxicity in treated cells. RAW264.7 cells were exposed to the HE, DCM, or EA fractions and subsequently incubated in a 96-well plate for 24 h. After incubation, an MTT solution (1 mg/ml) was added to each well, followed by an additional 4-h incubation period. The resulting formazan crystals were then dissolved by adding DMSO, and the mixture was allowed to react for 10 min. Finally, absorbance at 570 nm was measured using a UV/visible spectrophotometer (SpectraMax M2, Molecular Devices) to determine cell viability.

### Reverse Transcription Polymerase Chain Reaction (RT-PCR)

Following a 2-h pre-treatment with *P. citrinopileatus* fractions, RAW 264.7 cells seeded in 12-well plates were stimulated with LPS (1 μg/ml) for 24 h. Cellular total RNA was extracted with the RNeasy Mini Kit (Qiagen), which was then quantified. Complementary DNA (cDNA) was generated from 1 μg of total RNA with the Verso cDNA Kit (Thermo Fisher Scientific). PCR amplification was conducted with the PCR Master Mix Kit (Promega) utilizing primers specific to iNOS. iNOS primer sequences used for gene amplification in this study were listed below: iNOS mRNA: Forward: 5’-GTTACCATGAGGCTGAAATCC-3’, Reverse: 5’-CCTCTTGTCTTTGACCCAGTAC-3’; GAPDH mRNA: Forward: 5’-GGACCTCATGGCCTACATGG-3’, Reverse: 5’-TAGGGCCTCTCTTGCTCAGT-3’. After PCR amplification, products were resolved by agarose gel electrophoresis and analyzed for band intensity using UN-SCAN-IT gel software (version 5.1, Silk Scientific Inc.).

### Isolation of Nuclear Protein from RAW264.7 Cells

Upon completion of treatment, nuclear proteins were extracted using the Nuclear Extract Kit (Active Motif) in accordance with the manufacturer’s instructions. Briefly, cells were collected in ice-cold 1×hypotonic buffer and incubated on ice for 15 min. A detergent solution was then added, and the mixture was gently vortexed for 10 sec. The samples were centrifuged at 14,000 ×*g* for 1 min at 4°C, and the resulting pellet was retained for nuclear protein extraction. The pellet was resuspended in lysis buffer and subjected to gentle agitation at 4°C for 30 min to enhance protein solubilization. Following this, the lysate was centrifuged at 14,000 ×*g* for 10 min at 4°C, and the supernatant containing nuclear proteins was carefully collected. The extracted nuclear proteins were stored at -80°C until further analysis.

### SDS-PAGE and Western Blot Analysis

The culture medium was aspirated after treatment, and cells were gently washed twice with ice-cold PBS. Cell lysis for protein extraction was performed with RIPA buffer containing a protease and phosphatase inhibitor cocktail. Quantification of total protein was determined with the BCA Protein Assay Kit (Thermo Fisher Scientific). Electrophoretic separation of proteins was carried out on a 10% SDS-PAGE gel under 150 V and 400 A for 1 h. After electrophoresis, protein transfer onto a nitrocellulose membrane (Thermo Fisher Scientific) was carried out at 100 V and 300 A for 2 h. Blocking was performed with 5% nonfat milk for 1 h at room temperature with gentle shaking to reduce nonspecific interactions. The blocked membranes were exposed to primary antibodies (1:1,000 dilution) at 4°C for 16 h. The membrane underwent multiple TBST washes, followed by a 1-h incubation with 1:1,000 diluted secondary antibodies at room temperature. Protein bands were visualized using enhanced chemiluminescence (ECL) reagents (Amersham Biosciences Corp.) and imaged with the LI-COR C-DiGit Blot Scanner (LI-COR Biosciences). Band signal intensities were measured using UN-SCAN-IT gel software (version 5.1, Silk Scientific Inc.).

### Statistical Analysis

All experiments were conducted independently at least three times. Statistical analyses were performed using GraphPad Prism software (version 5.0, GraphPad Software, Inc.). Data are expressed as the mean ± standard deviation (SD). For statistical analysis, one-way ANOVA and Bonferroni’s post hoc test were employed to compare group means. Statistical significance was defined as a *p*-value below 0.05.

## Results and Discussion

### Effect of *Pleurotus citrinopileatus* on the NO Production and iNOS Expression in LPS-Stimulated RAW264.7 Cells

**Fig. 1:**
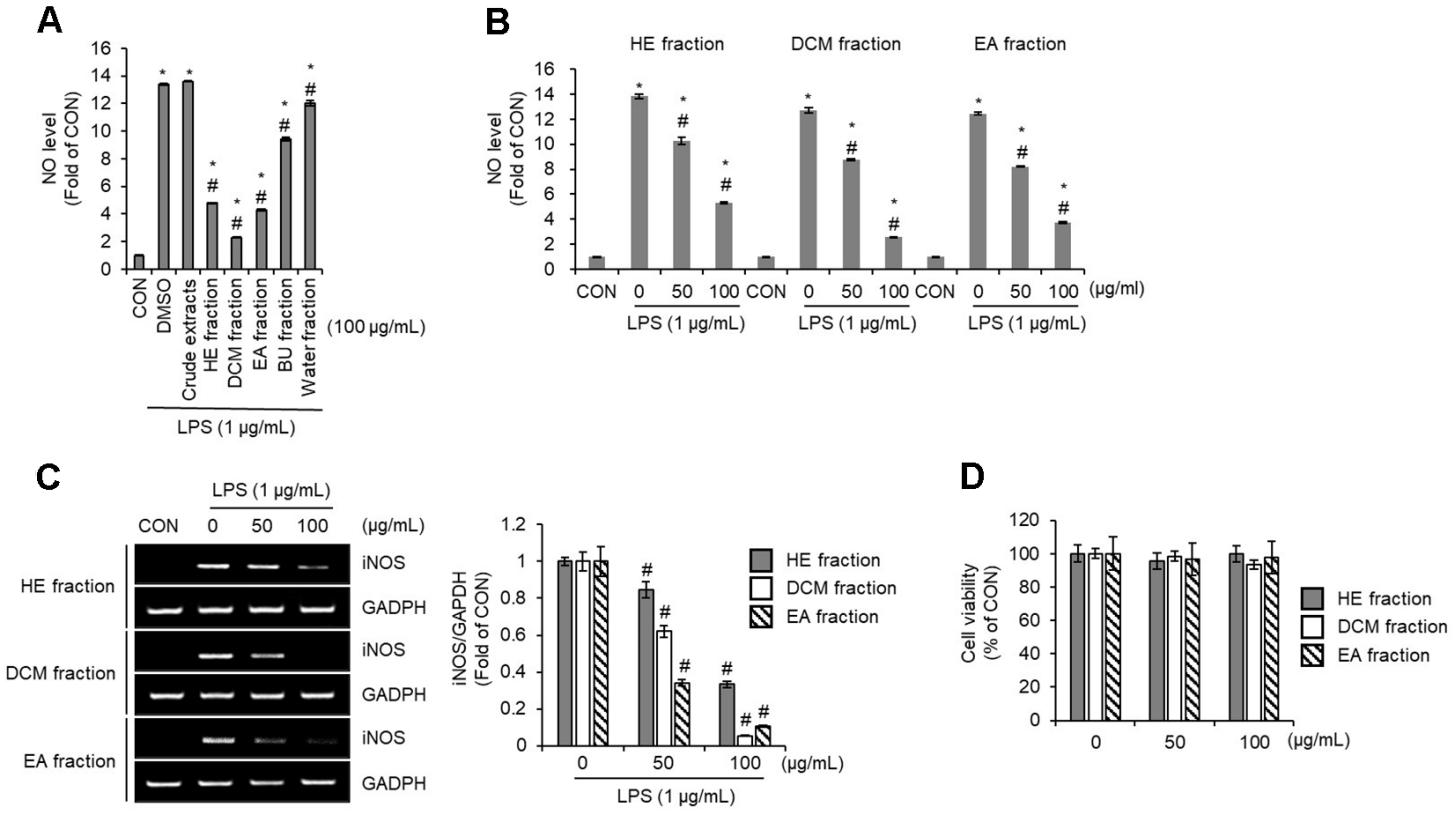
Effect of *Pleurotus citrinopileatus* on the NO production and iNOS expression in LPS-stimulated RAW264.7 cells. (**A**) RAW264.7 cells were pre-treated with crude extracts, HE fraction, DCM fraction, EA fraction, BU fractions, or water fraction from *Pleurotus citrinopileatus* for 2 h and then co-treated with LPS (1 μg/ml) for 24 h. NO level was measured by the Griess assay. (**B**) RAW264.7 cells were pre-treated with HE fraction, DCM fraction, or EA fraction of *Pleurotus citrinopileatus* for 2 h and then co-treated with LPS (1 μg/ml) for 24 h. NO level was measured by the Griess assay. (**C**) RAW264.7 cells were pre-treated with HE fraction, DCM fraction, or EA fraction of *Pleurotus citrinopileatus* for 2 h and then co-treated with LPS (1 μg/ml) for 24 h. mRNA level was measured by RT-PCR. (**D**) RAW264.7 cells were pre-treated with HE fraction, DCM fraction, or EA fraction of *Pleurotus citrinopileatus* for 24 h. Cell viability was measured using MTT assay. Data are presented as the mean ± standard deviation (*n* = 3). *P < 0.05 vs CON (untreated group). #*P* < 0.05 vs DMSO (LPS-only treated group).

Under normal physiological conditions, appropriately produced nitric oxide (NO) exerts beneficial effects in the human body, such as anti-inflammatory activity, acting as a potent neurotransmitter in neuronal synapses, and enhancing innate and adaptive immune systems. However, under pathological conditions, excessive NO production is known to play a pivotal role in the pathogenesis of inflammatory diseases [21]. Therefore, suppressing excessive NO production is considered a crucial therapeutic molecular target in treating inflammatory diseases [21]. In practice, the inhibition of NO production is widely utilized as a direct indicator of anti-inflammatory activity, owing to the close correlation between excessive NO generation and the induction of pro-inflammatory cytokines [22]. To evaluate the anti-inflammatory activity of *P. citrinopileatus*, we analyzed the extent to which the crude extract and various fractions of *P. citrinopileatus* inhibit LPS-induced NO production. The results revealed that the crude extract did not suppress LPS-induced NO production. However, the HE, DCM, and EA fractions significantly inhibited NO production, whereas the BU and water fractions exhibited only weak inhibitory effects (Fig. 1A). These findings suggest that the active compounds responsible for inhibiting NO production are predominantly concentrated in the n-hexane, dichloromethane, and ethyl acetate fractions. As such, these fractions are likely to serve as critical sources of anti-inflammatory bioactive compounds derived from *P. citrinopileatus*. The lack of activity in the crude extracts could be due to the presence of these active compounds in low concentrations, which became enriched through fractionation. The weak activity observed in the BU and water fraction indicates that polar compounds contribute only minimally to NO inhibition. Based on these findings, subsequent studies focused on the HE, DCM, and EA fractions of *P. citrinopileatus*. Because inducible nitric oxide synthase (iNOS) is responsible for NO production, the regulation of iNOS is also considered a key strategy in the management of inflammatory diseases [23]. As shown in Figs. 1B and 1C, the HE, DCM, and EA fractions dose-dependently inhibited LPS-induced NO production and iNOS expression in RAW264.7 cells. Moreover, the HE, DCM, and EA fractions exhibited no detectable impact on the viability of RAW264.7 cells (Fig. 1D). These findings suggest that the HE, DCM, and EA fractions suppress NO production by downregulating iNOS expression.

### Effect of HE, DCM, and EA Fraction of *Pleurotus citrinopileatus* on the MAPK and NF-κB Signaling Pathway in LPS-Stimulated RAW264.7 Cells

**Fig. 2:**
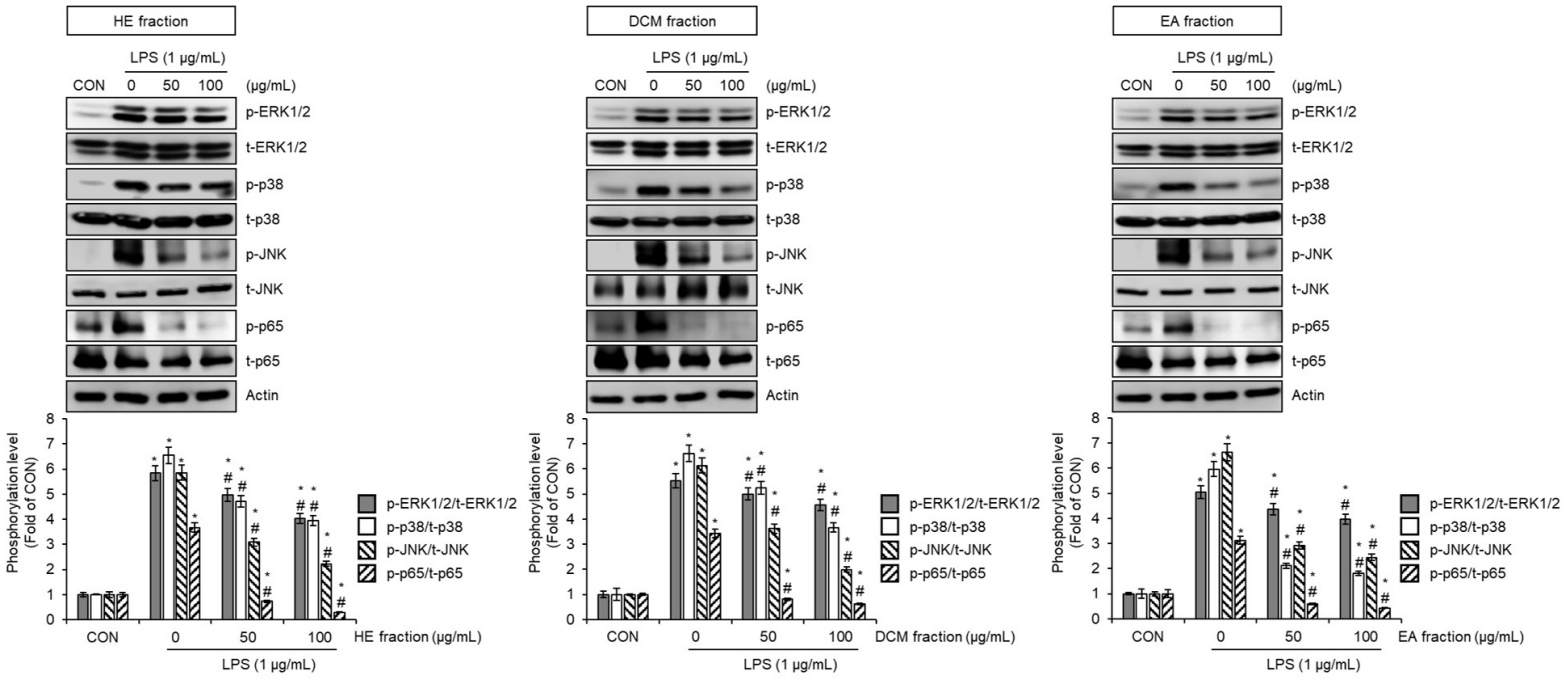
Effect of HE, DCM, and EA fraction of *Pleurotus citrinopileatus* on the MAPK and NF-κB signaling pathway in LPS-stimulated RAW264.7 cells. RAW264.7 cells were pre-treated with HE fractions, DCM fraction, or EA fraction of *Pleurotus citrinopileatus* for 2 h and then co-treated with LPS (1 μg/ml) for 30 min. The protein level was measured by Western blot analysis. Data are presented as the mean ± standard deviation (*n* = 3). *P < 0.05 vs CON (untreated group). #*P* < 0.05 vs cells treated with LPS alone.

To examine the role of mitogen-activated protein kinase (MAPK) and nuclear factor kappa B (NF-κB) signaling pathways in the suppression of nitric oxide (NO) production and inducible nitric oxide synthase (iNOS) expression by HE, DCM, and EA fractions in lipopolysaccharide (LPS)-stimulated RAW264.7 cells, we assessed whether these fractions influence LPS-induced phosphorylation of key signaling molecules. As illustrated in Fig. 2, treatment with the HE, DCM, and EA fractions resulted in a marked decrease in the phosphorylation levels of extracellular signal-regulated kinases 1/2 (ERK1/2), p38, c-Jun N-terminal kinase (JNK), and the NF-κB subunit p65 compared to cells exposed to LPS alone. These findings indicate that the anti-inflammatory effects of these fractions are closely linked to their capacity to interfere with LPS-triggered MAPK and NF-κB activation. Previous studies have demonstrated that inhibiting the MAPK cascade, including ERK1/2, p38, and JNK, effectively reduces inflammation in various in vivo models using mice and rats [24, 25]. Additionally, NF-κB activation is a well-established mechanism that amplifies inflammatory mediator production, contributing to the progression of chronic inflammation [26]. Given that both pathways play pivotal roles in inflammatory responses, their concurrent suppression is recognized as a valuable strategy for developing anti-inflammatory agents [24-26]. The ability of HE, DCM, and EA fractions to simultaneously modulate both MAPK and NF-κB pathways highlights their potential as multi-target regulators of inflammation. Overall, these findings suggest that the HE, DCM, and EA fractions effectively mitigate LPS-induced inflammatory responses by jointly targeting the MAPK and NF-κB pathways. This dual inhibition likely accounts for their ability to suppress NO production and iNOS expression, underscoring their potential therapeutic relevance in managing inflammation-related disorders.

### Effect of HE, DCM, and EA Fraction of *Pleurotus citrinopileatus* on Nrf2/HO-1 Signaling Pathway in RAW264.7 Cells

**Fig. 3:**
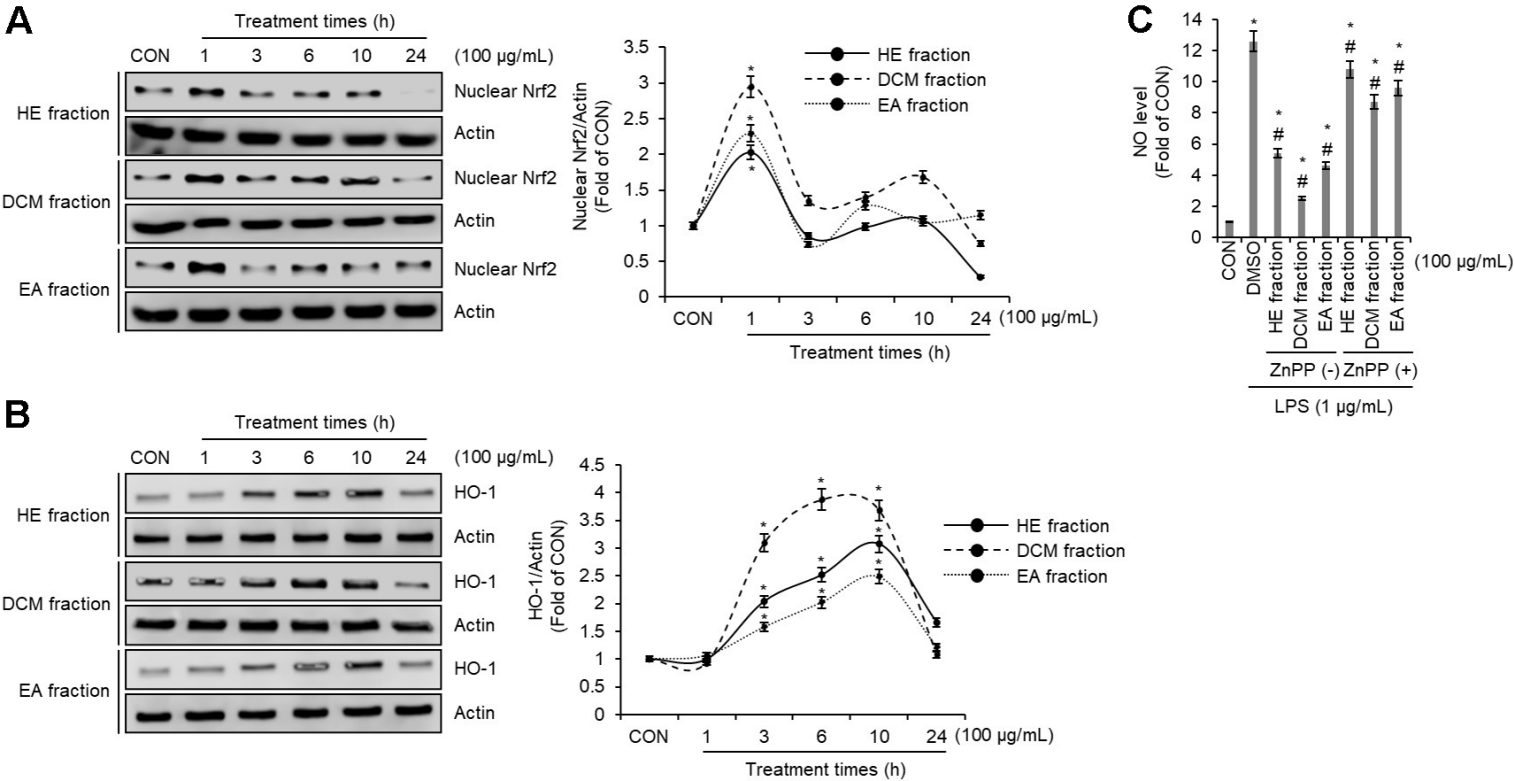
Effect of HE, DCM, and EA fraction of *Pleurotus citrinopileatus* on Nrf2/HO-1 signaling pathway in RAW264.7 cells. (**A**) RAW264.7 cells were treated with HE fraction, DCM fraction, or EA fraction of *Pleurotus citrinopileatus* for the indicated times. The protein level was measured by Western blot analysis. (**B**) RAW264.7 cells were treated with HE fraction, DCM fraction, or EA fraction of *Pleurotus citrinopileatus* for the indicated times. Nuclear fraction was prepared using Nuclear Extract Kit. The protein level was measured by Western blot analysis. (**C**) RAW264.7 cells were pretreated with HE fraction, DCM fraction, or EA fraction of *Pleurotus citrinopileatus* in the absence or presence of ZnPP (30 μM) for 2 h and then co-treated with LPS (1 μg/ml) for 24 h. NO level was measured by the Griess assay. Data are presented as the mean ± standard deviation (*n* = 3). *P < 0.05 vs CON (untreated group). #*P* < 0.05 vs DMSO (LPS-only treated group).

As a master regulator of oxidative stress response, nuclear factor erythroid 2-related factor 2 (Nrf2) orchestrates the expression of genes involved in cellular protection [27]. Under basal conditions, Nrf2 remains sequestered in the cytoplasm by Kelch-like ECH-associated protein 1 (Keap1), which acts as its negative regulator. Following oxidative stress, Nrf2 is released from Keap1 and migrates into the nucleus where it activates cytoprotective genes like heme oxygenase-1 (HO-1) by binding to the antioxidant response element (ARE) [28]. The anti-inflammatory role of Nrf2, particularly in sepsis-induced models, has been well established in prior studies. Notably, Nrf2-deficient mice display heightened inflammatory responses following LPS exposure, emphasizing its crucial function in mitigating inflammatory damage [29]. Additionally, HO-1, a downstream target of Nrf2, has been shown to mediate anti-inflammatory responses in RAW264.7 macrophages by suppressing the production of LPS-induced inflammatory mediators [30]. The findings imply that the Nrf2/HO-1 axis contributes both to oxidative stress protection and to the modulation of inflammatory pathways. To determine whether the suppression of NO production and iNOS expression by HE, DCM, and EA fractions involves the Nrf2/HO-1 pathway, we examined their impact on nuclear Nrf2 and HO-1 protein levels. As depicted in Figs. 3A and 3B, cells treated with these fractions exhibited a significant increase in nuclear Nrf2 and HO-1 protein expression compared to untreated controls. These results indicate that the HE, DCM, and EA fractions effectively activate the Nrf2/HO-1 pathway in RAW264.7 cells. Furthermore, the functional role of HO-1 in this process was confirmed by treating cells with zinc (II) protoporphyrin IX (ZnPP), a selective HO-1 inhibitor. As shown in Fig. 3C, ZnPP treatment attenuated the NO-suppressing effects of the HE, DCM, and EA fractions, suggesting that HO-1 induction is essential for their anti-inflammatory activity. Overall, these findings demonstrate that the HE, DCM, and EA fractions modulate inflammatory responses by activating the Nrf2/HO-1 signaling cascade. Their ability to enhance cellular defense mechanisms and suppress excessive inflammation underscores their potential as promising therapeutic candidates for managing inflammation and oxidative stress-related disorders. Furthermore, these insights lay the groundwork for future research into the therapeutic applications of HE, DCM, and EA fractions in inflammation-associated conditions.

### Effect of PI3K on the Activation of Nrf2/HO-1 Signaling Pathway by HE, DCM, and EA Fraction of *Pleurotus citrinopileatus* in RAW264.7 Cells

**Fig. 4:**
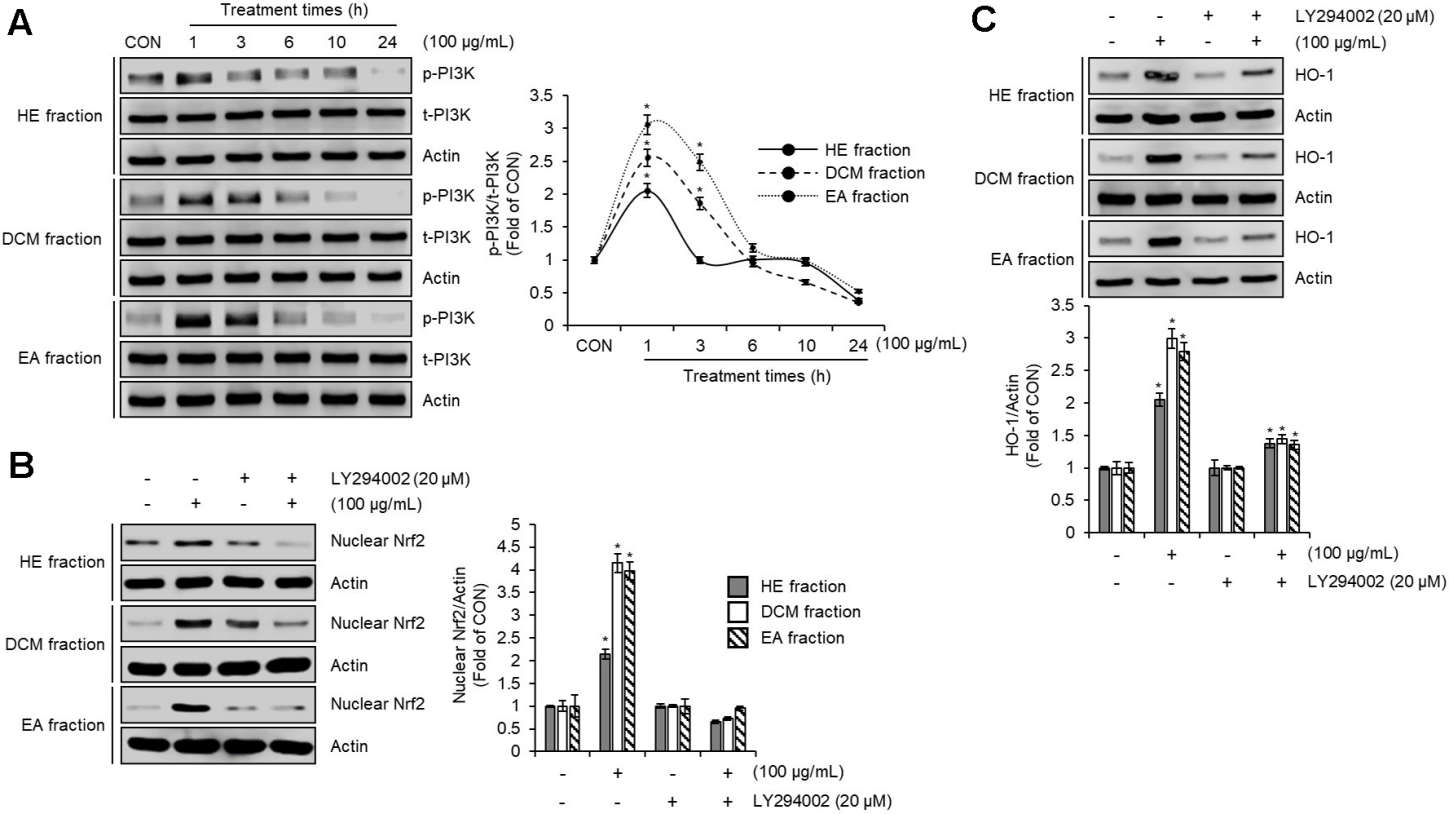
Effect of PI3K on the activation of Nrf2/HO-1 signaling pathway by HE, DCM, and EA fraction of *Pleurotus citrinopileatus* in RAW264.7 cells. (**A**) RAW264.7 cells were treated with HE fraction, DCM fraction, or EA fraction of *Pleurotus citrinopileatus* for the indicated times. (**B**) RAW264.7 cells were pre-treated with LY294002 for 2 h and the co-treated with HE fraction, DCM fraction, or EA fraction of *Pleurotus citrinopileatus* for 1 h. Nuclear fraction was prepared using Nuclear Extract Kit. (**C**) RAW264.7 cells were pre-treated with LY294002 for 2 h and the co-treated with HE fraction, DCM fraction, or EA fraction of *Pleurotus citrinopileatus* for 6 h. The protein level was measured by Western blot analysis. Data are presented as the mean ± standard deviation (*n* = 3). *P < 0.05 vs CON (untreated group).

Phosphoinositide 3-kinase (PI3K) has been reported to play a critical role in regulating the activation of the Nrf2/HO-1 signaling pathway, a key mechanism for protecting cells against oxidative stress and inflammation [31]. To investigate whether PI3K contributes to the activation of the Nrf2/HO-1 signaling pathway mediated by the HE, DCM, and EA fractions, we examined whether these fractions activate PI3K and whether inhibition of PI3K attenuates the activation of the Nrf2/HO-1 pathway induced by these fractions. As shown in Fig. 4A, treatment with HE, DCM, and EA fractions increased PI3K phosphorylation. The observed increase in PI3K phosphorylation upon treatment with these fractions confirms their ability to activate PI3K. Furthermore, inhibition of PI3K by LY294002 reduced the HE, DCM, and EA fraction-induced increases in nuclear Nrf2 and HO-1 protein levels compared to cells treated with the fractions in the absence of PI3K inhibition (Figs. 4B and 4C). These findings demonstrate the essential role of PI3K as an upstream regulator in the activation of the Nrf2/HO-1 signaling pathway. In conclusion, the ability of the HE, DCM, and EA fractions to activate PI3K, leading to the downstream activation of the Nrf2/HO-1 pathway, highlights their potential as promising therapeutic agents in the treatment of oxidative stress and inflammation-related diseases.

### Effect of ROS on the Activation of PI3K/Nrf2/HO-1 Signaling Pathway by HE, DCM, and EA Fraction of *Pleurotus citrinopileatus* in RAW264.7 Cells

**Fig. 5:**
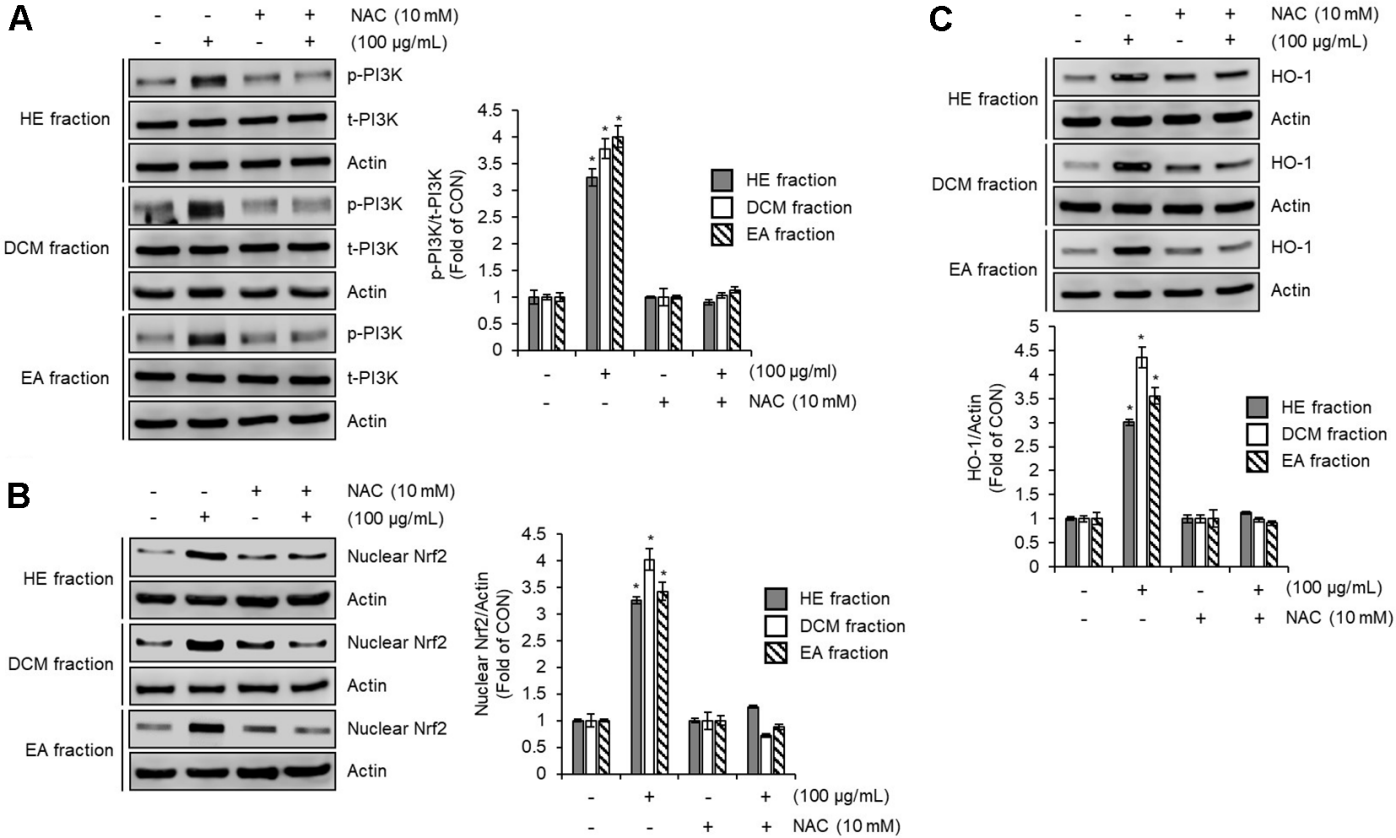
Effect of ROS on the activation of PI3K/Nrf2/HO-1 signaling pathway by HE, DCM, and EA fraction of *Pleurotus citrinopileatus* in RAW264.7 cells. (**A**) RAW264.7 cells were pre-treated with NAC for 2 h and co-treated with HE fraction, DCM fraction, or EA fraction of *Pleurotus citrinopileatus* for 1 h. (**B**) RAW264.7 cells were pre-treated with NAC for 2 h and co-treated with HE fraction, DCM fraction, or EA fraction of *Pleurotus citrinopileatus* for 1 h. Nuclear fraction was prepared using Nuclear Extract Kit. (**C**) RAW264.7 cells were pre-treated with LY294002 for 2 h and the co-treated with HE fraction, DCM fraction, or EA fraction of *Pleurotus citrinopileatus* for 6 h. The protein level was measured by Western blot analysis. Data are presented as the mean ± standard deviation (*n* = 3). *P < 0.05 vs CON (untreated group).

Reactive oxygen species (ROS) play a pivotal role in regulating the Nrf2/HO-1 signaling pathway, as previously documented [32, 33]. Additionally, ROS have been identified as upstream modulators of phosphoinositide 3-kinase (PI3K) [34], suggesting their involvement in the regulation of the PI3K/Nrf2/HO-1 signaling axis. Given these findings, we investigated whether ROS contribute to the activation of this pathway in response to HE, DCM, and EA fractions. To assess this, we examined the impact of ROS inhibition on the fraction-mediated phosphorylation of PI3K, as well as nuclear Nrf2 and HO-1 protein levels. As shown in Fig. 5A, treatment with N-acetyl-L-cysteine (NAC), an ROS scavenger, significantly reduced PI3K phosphorylation induced by the HE, DCM, and EA fractions. This suppression of PI3K activation was accompanied by a decrease in nuclear Nrf2 and HO-1 protein expression (Figs. 5B and 5C), demonstrating the crucial role of ROS as upstream regulators in the PI3K/Nrf2/HO-1 pathway. Furthermore, previous studies have indicated that ROS can also modulate NF-κB signaling by exerting inhibitory effects on its activation [35]. Although this study did not directly assess the influence of ROS on NF-κB inhibition mediated by the HE, DCM, and EA fractions, the findings suggest that ROS may contribute to their anti-inflammatory action through multiple pathways, including both PI3K/Nrf2/HO-1 activation and potential NF-κB suppression.

Edible mushrooms are widely recognized for their bioactive compounds, including polysaccharides, phenolic derivatives, indolic compounds, mycosteroids, fatty acids, carotenoids, vitamins, and biometals, which contribute to their anti-inflammatory properties [36]. Among these, many compounds have been reported to suppress inflammation by inhibiting NF-κB signaling [36]. Notably, mushrooms from the *Pleurotus* genus have been shown to attenuate NF-κB activation, reinforcing their anti-inflammatory potential [37]. Consistent with these findings, our study demonstrates that the HE, DCM, and EA fractions of *P. citrinopileatus* effectively downregulate NF-κB signaling, aligning with the well-established anti-inflammatory mechanisms of edible mushrooms. Additionally, our results expand on prior studies by showing that these fractions also inhibit the MAPK signaling pathway, further contributing to their inflammation-suppressing effects. A key distinction of this study is the identification of an alternative anti-inflammatory mechanism mediated by ROS/PI3K/Nrf2/HO-1 signaling activation. While previous research on *Pleurotus* species has predominantly focused on NF-κB inhibition, our findings suggest that *P. citrinopileatus* exerts a dual regulatory effect. Specifically, it not only suppresses pro-inflammatory signaling through NF-κB and MAPK inhibition but also enhances antioxidant and cytoprotective responses via the PI3K/Nrf2/HO-1 axis. This integrated mechanism results in reduced iNOS expression and subsequent NO suppression, further supporting the potential therapeutic value of *P. citrinopileatus* in inflammation-associated conditions.

However, this study has several limitations. First, while the HE, DCM, and EA fractions demonstrated anti-inflammatory activity, the specific bioactive compounds responsible for these effects were not identified. Further investigations are required to isolate and characterize the active components within these fractions to gain a deeper understanding of their mechanisms of action. Previous research has identified a nonlectin glycoprotein from *P. citrinopileatus* that inhibits iNOS expression and NO production via NF-κB suppression in LPS-stimulated RAW264.7 cells [38]. Thus, we cannot rule out the possibility that nonlectin glycoproteins contribute to the observed anti-inflammatory effects of the HE, DCM, and EA fractions. Second, as this study was conducted in vitro, its findings may not fully translate to in vivo conditions. To confirm the proposed anti-inflammatory mechanisms, additional in vivo studies using animal models are necessary. These studies will help clarify the therapeutic potential and safety profile of the HE, DCM, and EA fractions. Despite these limitations, this study provides valuable insights into the anti-inflammatory mechanisms of *P. citrinopileatus* and serves as a foundation for future research aimed at exploring its potential in therapeutic applications.
